# Tuber Cinereum Diverticula in a 28-Month-Old with Xq21 Deletion Syndrome

**DOI:** 10.1155/2014/413574

**Published:** 2014-07-13

**Authors:** Matthew T. Whitehead, Gilbert Vezina

**Affiliations:** Department of Radiology, Children's National Medical Center, 111 Michigan Avenue, NW, Washington, DC 20010, USA

## Abstract

A developmentally delayed 28-month-old male toddler was referred to us for brain MRI. Imaging revealed corpus callosum dysgenesis, forniceal hypoplasia, vermian hypoplasia, and hypothalamic dysmorphism characterized by tuber cinereum diverticula. Subsequent chromosomal microarray showed an Xq21 deletion. We present a case of Xq21 deletion syndrome with midline brain anomalies and a novel hypothalamic malformation.

## 1. Introduction

Xq21 deletion syndrome comprises intellectual disability, choroideremia, and deafness. By strict definition, defects affecting both CHM and POU3F4 genes must be present [1]. CHM gene mutations cause choroideremia. Disruptions or mutations of the POU3F4 gene have been implicated in X-linked deafness-2 (DFNX2), also called conductive deafness with stapes fixation (DFN3). From an imaging standpoint, cochlear malformations have been demonstrated previously [2, 3]. Brain parenchymal abnormalities in Xq21 deletion syndrome have not been described to the best of our knowledge. We present a case of a 28-month-old male with developmental delay, found to have an Xq21 deletion and midline brain anomalies including peculiar tuber cinereum diverticula.

## 2. Case Presentation

A 28-month-old male toddler underwent workup for global developmental delay and mild hypotonia. He was the product of an uncomplicated pregnancy, born at term via C-section due to concern for possible cephalopelvic insufficiency. The parents were nonconsanguineous and had no significant past medical history. The patient was of normal height and weight for age; body mass index was at the 68th percentile. Ophthalmic examination performed for evaluation of intermittent exotropia was otherwise normal. A neonatal otoacoustic emissions hearing exam was also normal. Ultimately, chromosomal microarray revealed a pathogenic Xq21.1q21.2 chromosome deletion involving the following genes: POU3F4, CHM, POF1B, RPS6KA6, HDX, UBE2DNL, APOOL, SATL1, ZNF711, and DACH2. During the course of the workup, the patient was referred to our imaging service to exclude congenital intracranial abnormalities.

Brain imaging was performed on a 1.5T MR (Signa HDxt Optima edition, General Electric, Milwaukee, WI). Prescribed pulse sequences included Sagittal T1WI SPGR (Spoiled Gradient Echo), axial T2WI, axial fat saturated T2 FLAIR (Fluid Attenuation Inversion Recovery), axial diffusion weighted images, axial SWAN (Susceptibility-Weighted Angiography), and coronal fat saturated T2WI.

**Figure 1:**
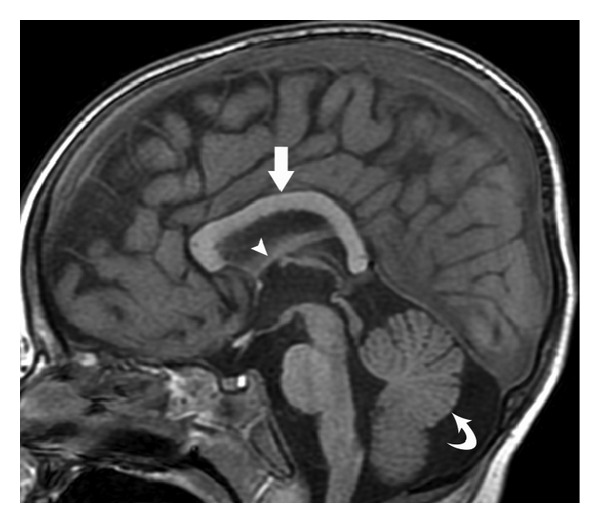
Sagittal SPGR T1WI (repetition time msec/echo time msec/inversion time msec, 11/5/500) showing a relatively featureless, mildly dysgenetic corpus callosum of uniform thickness (straight arrow), small fornices consistent with hypoplasia (arrowhead), and mild generalized vermian hypoplasia (curved arrow). Note normal pituitary gland.

**Figure 2:**
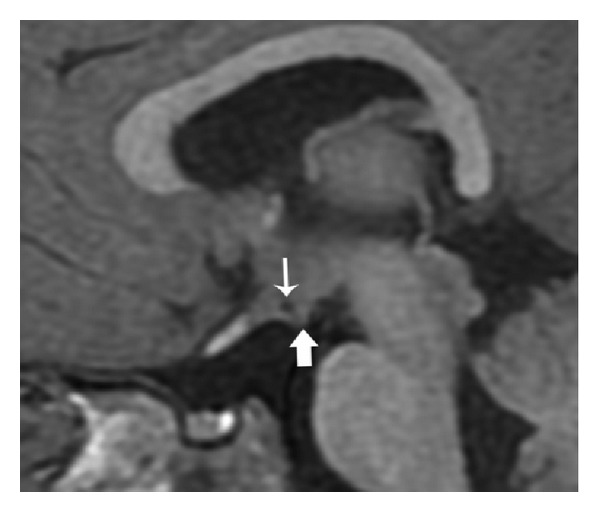
Parasagittal SPGR T1WI (repetition time msec/echo time msec/inversion time msec, 11/5/500) depicting a small diverticulum along the ventrolateral 3rd ventricular floor/tuber cinereum projecting into the suprasellar cistern (thick arrow). Note central hypointensity representing 3rd ventricular CSF within (thin arrow).

**Figure 3:**
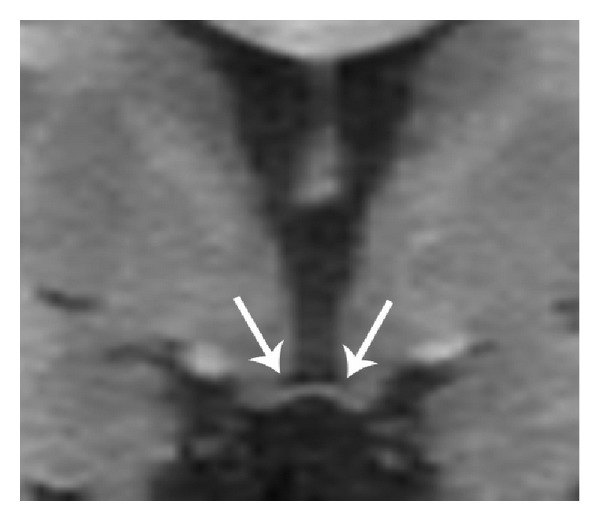
Reformatted coronal SPGR T1WI (repetition time msec/echo time msec/inversion time msec, 11/5/500) demonstrating small, broad based diverticula involving the tuber cinereum that contain hypointense 3rd ventricular cerebrospinal fluid (arrows). The 3rd ventricular floor is enlarged in the transverse dimension.

**Figure 4:**
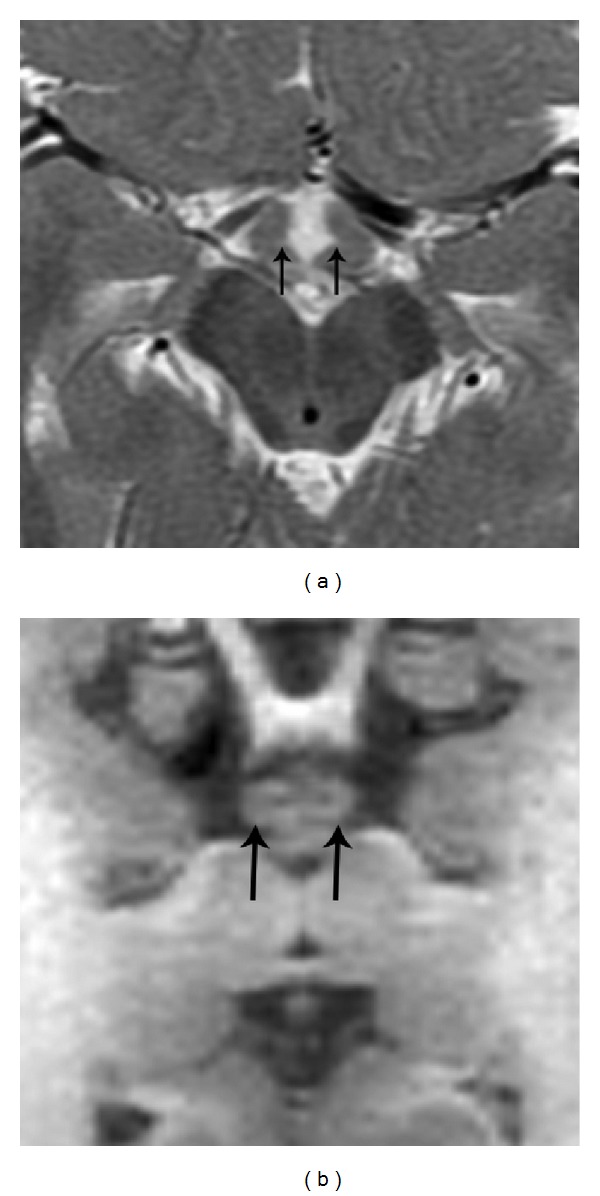
Axial T2WI (repetition time msec/echo time msec, 4400/103) (a) and reformatted oblique axial SPGR T1WI (repetition time msec/echo time msec/inversion time msec, 11/5/500) (b) demonstrating small, broad based diverticula involving the tuber cinereum that contain 3rd ventricular cerebrospinal fluid (arrows).

Midline sagittal images demonstrate corpus callosum dysgenesis, hypoplastic fornices, and vermian hypoplasia (Figure 1). Parasagittal, coronal, and axial images show unique maldevelopmental hypothalamic morphology: bilaterally symmetric diverticula extend from the ventrolateral surfaces of the tuber cinereum (Figures 2, 3, and 4). The floor of the third ventricle is mildly broadened. The pituitary gland is normal (Figure 1). The globes are normal. The membranous labyrinths are suboptimally evaluated due to inadequate slice thickness and, however, are without gross abnormality.

## 3. Discussion

Xq21 deletion is a rare genetic syndrome with phenotypic manifestations including intellectual disability, hearing loss, and vision loss. Intellectual disability is a common consequence of X chromosome aberrations; genetic defects in Xq12-Xq21 are often responsible [4]. Visual and hearing deficits result from involvement of the CHM and POU3F4 genes, respectively. Both of these genes are located on the long arm of the X chromosome and are deficient in Xq21 deletion syndrome. CHM gene defects can cause choroideremia, a progressive degeneration of the choroid and retina [5]. POU3F4 gene defects have been implicated in patients with congenital stapes fixation and labyrinthine dysplasia associated with sensorineural hearing loss [2, 3]. Although developmental delay and intellectual disability were present in our patient, auditory and ophthalmic exams failed to demonstrate abnormalities despite CHM and POU3F4 gene defects. However, serial follow-up ophthalmic exams are warranted in this case because visual deficits tend to be progressive over time [3]. Additionally, the patient will require auditory monitoring based on a strong predisposition to hearing loss known to be associated with POU3F4 gene defects. Our patient's newborn hearing screen was performed using the most commonly employed assessment, the otoacoustic emissions test (OAE). While the efficacy of OAE and auditory brainstem response test (the main alterative newborn screening exam) seems largely comparable, the exact sensitivities of these tests remain undetermined, and false negative OAE cases have been documented [6, 7]. It is notable that minor temporal bone, labyrinth, and cochlear nerve anomalies may be occult on our patient's brain MR that was not optimized to evaluate otologic structures. Furthermore, dedicated temporal bone imaging has not been pursued. We query whether delayed speech development in this child could be related to an undiagnosed hearing impairment.

**Figure 5:**
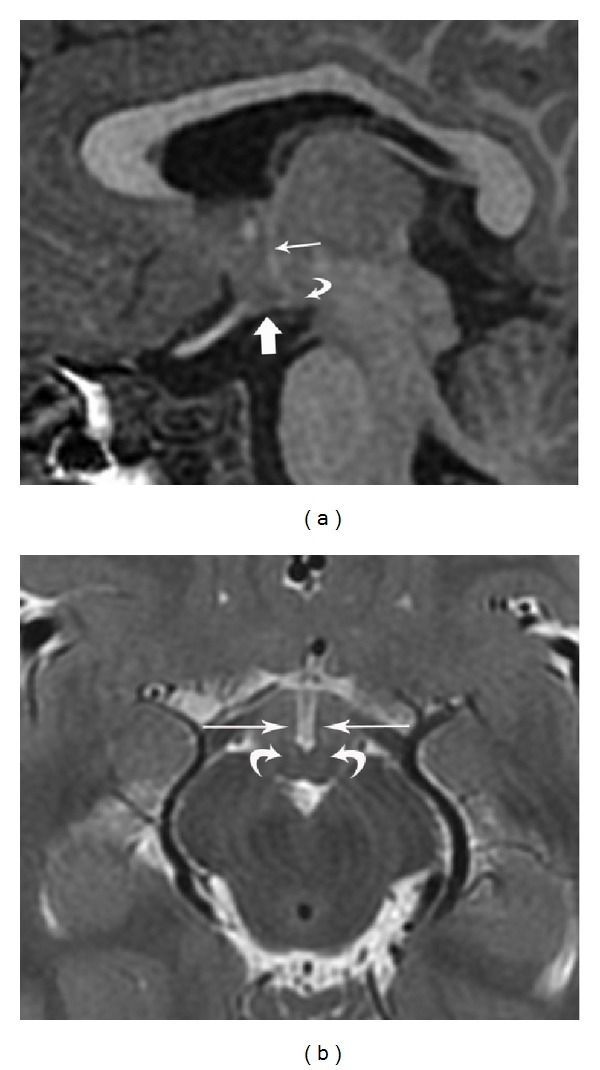
(a) Parasagittal SPGR T1WI (repetition time msec/echo time msec/inversion time msec, 8/3/450) from a 3-year-old male depicting normal brain anatomy. The lateral portion of the hypothalamic tuber cinereum has a straight outer contour (thick arrow), in contrast to the diverticulum shown in Figure 2. The normal mammillary body (curved arrow) and forniceal column (thin arrow) are partially visible. (b) Axial T2WI (repetition time msec/echo time msec, 3200/111) from a 3-year-old male showing normal hypothalamic anatomy. Hypothalami are visible near midline, marginating the 3rd ventricle (straight arrows). The inner margins of the hypothalami are uniformly straight apart from the rounded contour of the mammillary bodies (curved arrows).

The hypothalamus marginates most of the anteroinferior 3rd ventricle. The hypothalamic component of the 3rd ventricular floor extends from the pituitary infundibulum (anterior) to the mammillary bodies (posterior), with the tuber cinereum located in between [8] (Figure 5). The tuber cinereum contains two important nuclei: ventromedial and arcuate. Together with the adjacent lateral hypothalamic area, the ventromedial nuclei govern feeding behavior [9]. The ventromedial nuclei have earned the title “satiety center” based on the observation that lesions involving these nuclei can result in hyperphagia and obesity, aggressive behavior, and irritability. The nearby lateral hypothalamic area has been called the “feeding center” because lesions here can cause anorexia. The arcuate nucleus is also involved in feeding behavior and helps modulate endocrine functions in the adenohypophysis [10]. Despite our patient's structural tuber cinereum malformation, there were no signs or symptoms of endocrinopathy, obesity, or anorexia.

Brain MR revealed midline anomalies including dysgenesis of the corpus callosum, forniceal hypoplasia, vermian hypoplasia, and broadening of the 3rd ventricular floor. Additionally, a novel hypothalamic dysmorphology was discovered. Small diverticula were present extending from the ventrolateral margins of the tuber cinereum (Figures 2, 3, and 4). Neither the globes nor the membranous labyrinths demonstrated abnormality.

## 4. Conclusion

We discovered an unprecedented hypothalamic malformation characterized by ventrolateral tuber cinereum diverticula in a male toddler with Xq21 deletion. Additional midline brain anomalies including corpus callosum dysgenesis, forniceal hypoplasia, and vermian hypoplasia were coexistent.
